# Protocol for two models of behavioral transition from action to no-action when facing prolonged uncontrollable experience in mice

**DOI:** 10.1016/j.xpro.2024.102967

**Published:** 2024-03-15

**Authors:** Chaoqun Li, Ying Zhang, Tianping Sun, Nashat Abumaria

**Affiliations:** 1 State Key Laboratory of Medical Neurobiology and MOE Frontiers Center for Brain Science, Institutes of Brain Science, Fudan University, Shanghai 200032, China; 2 Department of Histology and Embryology, and Shanghai Key Laboratory of Cell Engineering, Naval Medical University, Shanghai 20043, China

**Keywords:** Model Organisms, Neuroscience, Cognitive Neuroscience, Behavior

## Abstract

Uncontrollability could lead to behavioral adjustment or even giving up when facing repeated failure. Here, we detail a protocol to study the behavioral transition from action to no-action induced by prolonged uncontrollable experiences in mice. We describe the behavioral devices, video analysis, and the exponential learning curve fitting for mathematical assessment. We perform further validation experiments evaluating locomotor, social, and anxiety-/depression-like behaviors. This approach helps study neural mechanisms underlying adaptive decision-making when facing repeated failure.

For complete details on the use and execution of this protocol, please refer to Li et al.^1^

## Highlights

• Steps for creating setups and exposing mice to prolonged uncontrollable experiences
• Steps for quantifying the behavioral transition from action to no-action in mice
• Steps for computational modeling based on the exponential learning equation
• Steps for performing validation experiments to exclude alternative interpretations

## Abstract (editor highlights)

Publisher’s note: Undertaking any experimental protocol requires adherence to local institutional guidelines for laboratory safety and ethics.

## Graphical abstract

**Figure:**
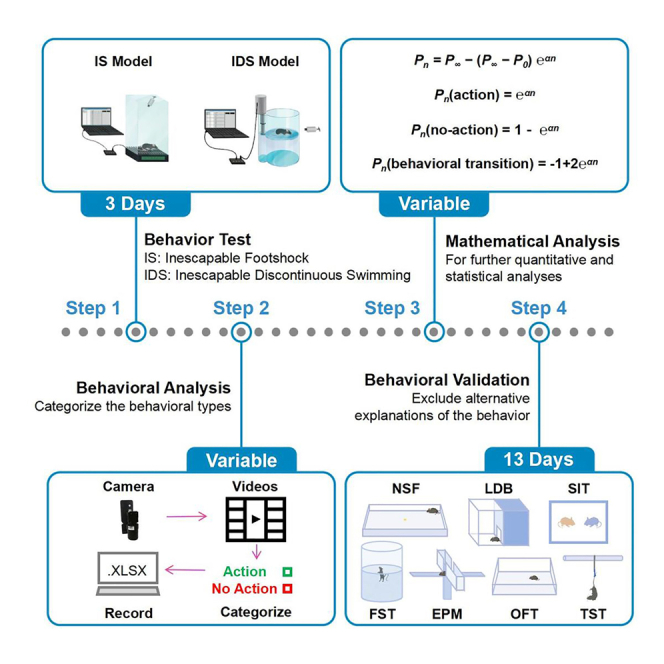

## Before you begin

Fight or flight will always be the first decision to make when individuals encounter stressful or threatening situations. However, individuals will gradually give up futile attempts if these situations are prolonged and uncontrollable. Neural mechanisms underlying this give up-/quitting-like adaptive behavior remain largely unknown. One of the challenges in this area is the lack of suitable animal models.

A cognitive psychology theory postulates that subjects show an action-oriented cognitive state once confronted with an uncontrollable experience. If uncontrollability is prolonged, the subjects gradually transition to a no-action-oriented cognitive state.^2^ Experimental studies in human subjects show that perceived controllability promotes persistence of behavior,^3^ whereas uncontrollability promotes quitting.^4^ Based on this rationale, we establish and validate two mouse models of behavioral transition from action to no-action by exposing mice to prolonged experiences with an uncontrollable outcome.

In the first model, we expose mice to three sessions (1 h/session, 1 session/day) of inescapable electrical foot shock (IS model). In the second model, we expose mice to three sessions (1 h/session, 1 session/day) of inescapable discontinuous swimming (IDS model). Each session is composed of 60 trials of uncontrollable experiences. We videotape the sessions and analyze the mouse behavior during the foot shock or swimming intervals.

### Protocol overview

This protocol will be a manual in which we describe a detailed approach to construct, analyze and further validate two animal models of behavioral transition from action to no-action induced by prolonged uncontrollable experiences in mice. The protocol consists of four major steps: i) setting up the two models (IS and IDS models), ii) analyzing the videos to determine the type of behavior during each trial (i.e., during foot shock or swimming period), iii) building a mathematical model of the behavioral transition and using it for quantitative and statistical analyses and iv) validating the models (model generalizability to male and female mice, and the behavioral transition is not associated with changes in locomotor/social/depression/anxiety-related behaviors).

### Institutional permissions

We use male/female adult C57BL/6J mice (2–4 months old, Jie Si Jie Laboratory Animals Shanghai, China). The mice are group-housed (3–5 mice/cage) under controlled temperature (23 ± 2°C), 12:12 h reversed light-dark cycle (light onset at 8:00 p.m.) with food and water ad libitum. All experiments are performed according to the guidelines of the Fudan University Committee for Animal Care and Use (license number: SYXK-2020-0032).

## Key resources table

**Table:** 

REAGENT or RESOURCE	SOURCE	IDENTIFIER
**Experimental models: Organisms/strains**		

Mouse: C57BL/6J, 2–4 months, male/female	Jie Si Jie Laboratory Animals Co., Ltd, Shanghai	C57BL/6JRj

**Software and algorithms**		

Graphic State 4	Coulbourn version 2.10.00	https://actimetrics.com/downloads/graphic-state/
Limelight 4	Coulbourn version 2.10.00	https://actimetrics.com/products/limelight/
GraphPad Prism 7.04	GraphPad Software	https://www.graphpad.com

**Other**		

Coulbourn Instruments H13-15 precision-regulated animal shocker	Coulbourn Instruments	N/A
Coulbourn Habitest H02-01 Habitest Linc	Coulbourn Instruments	N/A
Coulbourn H03-04 Habitest Linc output converter environment connection board	Coulbourn Instruments	N/A
Habitest operant cages	Coulbourn Instruments	N/A
High voltage modular shocker floor	Coulbourn Instruments	N/A
Coulbourn Habitest H02-01 Habitest Linc	Coulbourn Instruments	N/A
Coulbourn H03-04 Habitest Linc output converter environment connection board	Coulbourn Instruments	N/A
Stepper controller	Probecare Scientific, Wuhan, China	N/A
Linear actuator	Probecare Scientific, Wuhan, China	N/A
AC/DC adapter	Probecare Scientific, Wuhan, China	N/A

## Step-by-step method details

### Set up the two behavioral models

**Timing: 3 days for each model**

This section describes using inescapable foot shock or inescapable discontinuous swimming experience to build animal models of behavioral transition from action to no-action when facing prolonged uncontrollable experience in mice.

***Note:*** The behavioral testing room is kept under the following environmental conditions: temperature 23 ± 2°C, humidity 40%–60%, background noise 52 dB and red-dim light. All behavioral devices/setups are placed inside a light and sound insulating cabinet. The cabinet is equipped with a fan, which is turned off during the test resulting in reducing the background noise inside the cabinet to 42dB. Once the animal is placed in the behavioral device, the cabinet door remains closed until the end of the session. The experimenter remains in the same room to monitor the session through the computer screens beside the insulating cabinet. All animals are handled daily, 3–5 days before the experiment starts. Handling includes gently picking up the animal, placing it on the lap and returning it to the cage. Animals are brought to the behavioral testing area one day before the experiment to adapt to the environment and kept in a separate room all the time.

1. Setup the inescapable Foot Shock model (IS model).
  a. Use one side of the standard shuttle-box cage (Figure 1) to make the IS model.
    ***Note:*** This cage is connected to a computer through an environment connection board, shock generator, and camera (top) to control stimulus presentation, shock delivery, and record animal behavior (the whole apparatus, Figure 1A). The cage consists of two chambers, light and dark (Figure 1B). We use the light transparent chamber only to expose the animals to inescapable electrical foot shocks by keeping the middle door between the two chambers permanently closed. The chamber has two transparent acrylic walls (the front is a door). The other two sides are metal. The dimensions of the chamber are 17 × 17.5 × 28.5 cm (length × width × height). The floor has a metal grid for the electrical shock (15 metal grids). For a complete presentation of the components and apparatus, refer to the key resource table and Figure 1.

**Figure 1:**
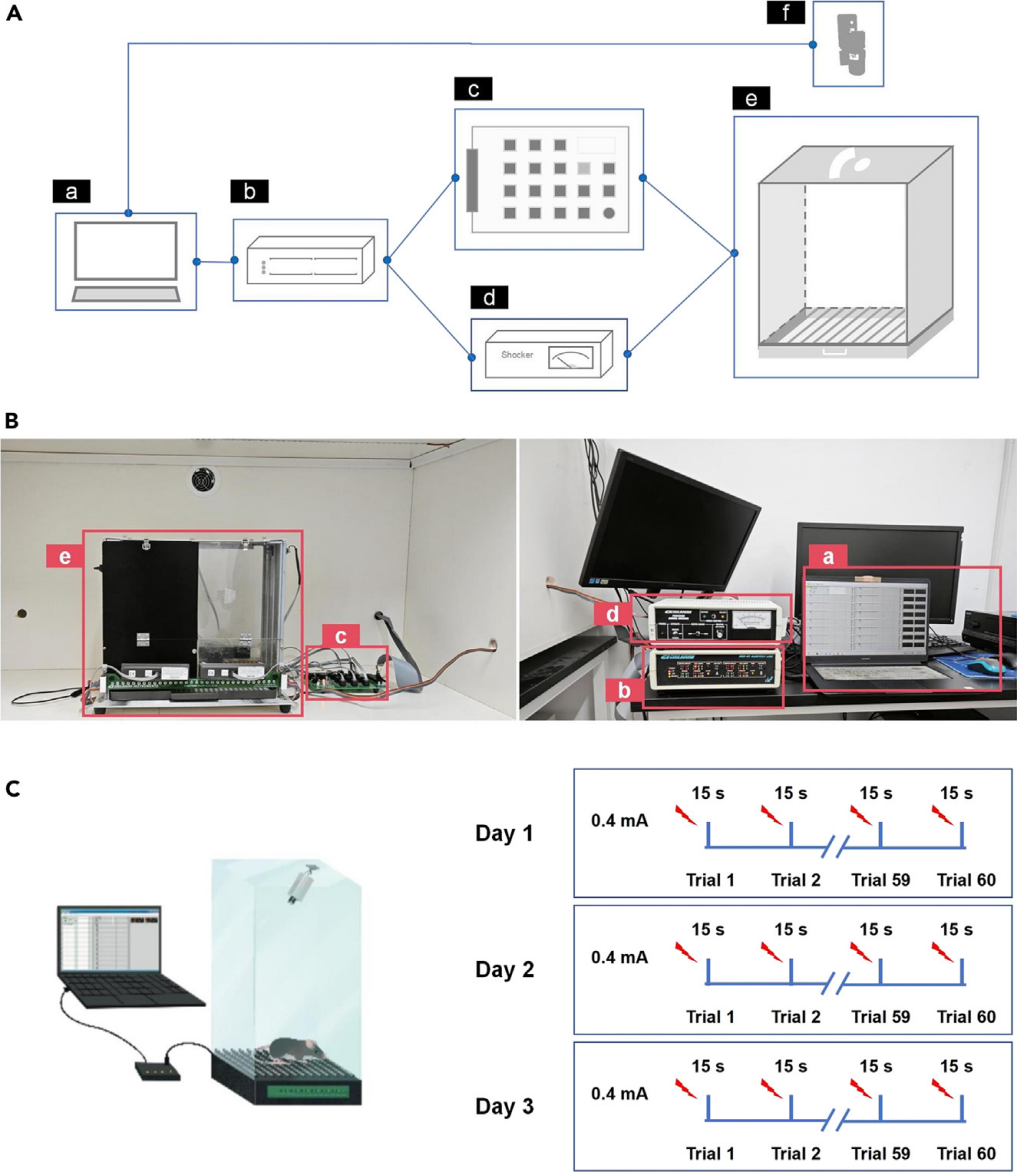
The assembly of the inescapable foot shock (IS) apparatus and the protocol of the IS model (A) The assembly diagram of the IS model apparatus is simplified to illustrate the logical hierarchical relationship between the components. Power connections are not shown. The components include: a. Computer, b. Coulbourn Habitest H02-01 Habitest Linc, c. Coulbourn H03-04 Habitest Linc Output Converter Environment Connection Board, d. Coulbourn Instruments H13-15 Precision-Regulated Animal Shocker, e. Inescapable shock chamber. The round hole at the top of the chamber is designed for the camera, and the slender hole is reserved for the fiber during *in vivo* recording. f. Camera. (B) Pictures display the components and their connections in real life. Components (a to e) correspond to the elements in (A). The computer (a) screen shows the Graphic State 4.0 software. The inescapable shock chamber (e) consists of two chambers, transparent and dark (we use the transparent chamber). (C) A simplified illustration of the IS apparatus and a diagram of the IS protocol.
  b. Utilize Graphic State 4.0 software to configure and execute the IS program. The IS experiment consists of three repetitive sessions conducted over three consecutive days. The program details for one session are as follows:
    i. Contain 60-trial number per session.
    ii. Before the first trial of each session, habituate the mice for 5 min.
    iii. Give a 15 s foot shock (0.4 mA) per trial.
    iv. Give a viable inter-trial interval between two consecutive trials (20–90 s, mean = 45 s).
      ***Note:*** The variability in the inter-trial interval is crucial for mice to perceive the foot shock as unpredictable and discontinuous.
  c. Run IS protocol (Figure 1C).
    i. Gently place a mouse into the foot shock cage.
    ii. Initiate the IS program and start video recording.
    iii. After each session, gently remove the mouse from the foot shock cage and return it to its home-cage.
    iv. Thoroughly clean the foot shock cage, remove any excretions, and wipe it with alcohol to minimize residual odors from the previous mouse.
    v. Repeat the same protocol on Days 2 and 3.
2. Setup the inescapable Discontinuous Swimming model (IDS model).
  a. Use IDS apparatus (Figure 2A, China patent, 2023) to make the IDS model.
    ***Note:*** To expose animals to the inescapable discontinuous swimming experience, we have developed, constructed, and assembled a new setup: the IDS cylinder made from transparent acrylic material (Figure 2B, Methods video S1). This cylinder is connected to a computer through an environment connection board, a stepper controller, and a camera (on the side) to control the robotic arm and record animal behavior (the whole apparatus, Figure 2A). We have integrated a robotic arm, with a rescue plate at its end, into a cylinder filled with water to leave the animal swimming in water for a short period (15 s) before taking it out of the water by the robotic arm and rescuing plate. By repeating these steps, we are exposing the mice to a prolonged, discontinuous swimming experience. The IDS cylinder (diameter = 19 cm, height = 41 cm) and arm holder (55 × 48 cm, height × width) are built on a 30 × 30 cm acrylic base. The robotic arm (55 cm from the base) has a rescuing plate attached to its end (16 cm, Figure 2B). The water will be placed inside the cylinder to a height of 21 cm. The rescue plate will be elevated and kept above the water surface at a height of 24 cm. During the discontinuous swimming trials, the rescue plate will be pushed down by the robotic arm (down to 11.5 cm from the base). For a complete presentation of the components and apparatus, see the key resource table and Figure 2 (also see Methods video S1). If a robotic arm is unavailable, a simple linear actuator can be purchased (online) and fitted with the computer system as described here. To reduce the contribution of human error, we do not recommend using the manually controlled system to descend and elevate the animal from the water or to determine the intertrial intervals (it is randomized).

**Figure 2:**
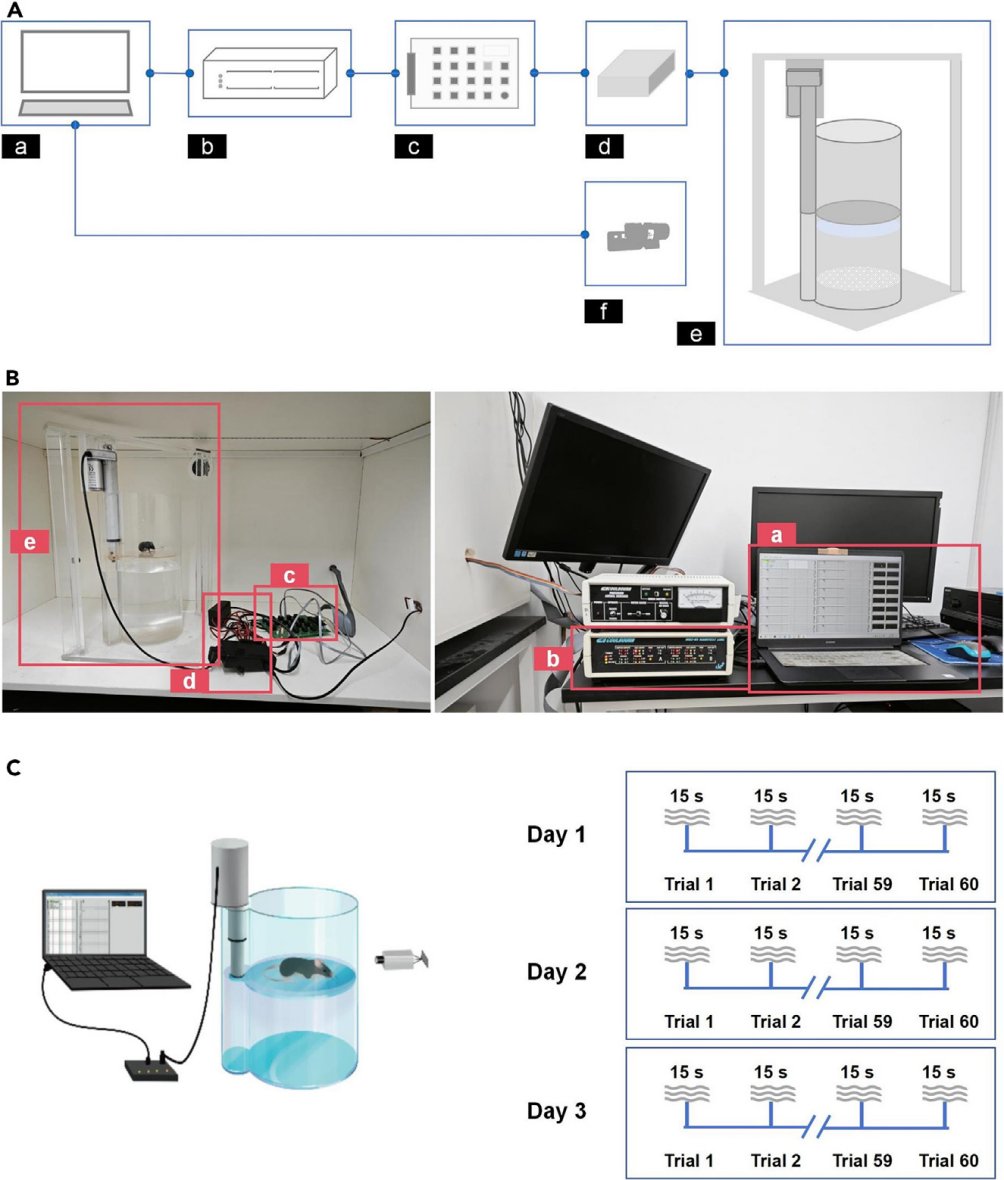
The assembly of the inescapable discontinuous swimming (IDS) apparatus and the protocol of the IDS model (A) The assembly diagram of the IDS apparatus is simplified to illustrate the logical hierarchical relationship between components. Power connections are not shown. The components include: a. Computer, b. Coulbourn Habitest H02-01 Habitest Linc, c. Coulbourn H03-04 Habitest Linc Output Converter Environment Connection Board, d. Stepper Controller and AC/DC adapter, e. We created the IDS novel device. (B) Pictures display the components and their connections in real life. Components (a to e) correspond to the components in (A). The computer (a) screen shows the Graphic State 4.0 software. (C) A simplified illustration of the IDS apparatus and a diagram of the IDS protocol. See also Methods video S1.
    Methods video S1. Illustrative video for the newly designed IDS device, related to step 2a To expose mice to an inescapable discontinuous swimming experience, we developed a new device composed of a transparent cylinder made of acrylic materials equipped with a robotic arm attached to a rescue plate. The robotic arm will drop down the rescue plate deep into the water, leaving the mouse (represented by a 50 mL tube) swimming before the arm rises, allowing the rescue plate to take the mouse out of the water. The robotic arm is connected to a computer via a programmable interface. The videos are adapted with permission from Li et al. (2023).^1^
  b. Utilize Graphic State 4.0 software to configure and execute the IDS program. The IDS experiment comprises three repetitive sessions conducted over three consecutive days. The program details for one session are as follows:
    i. Contain 60-trial number per session.
    ii. Before the first trial of each session, habituate the mice for 5 min.
    iii. Give a 15 s discontinuous swimming per trial.
    iv. Give a viable inter-trial Interval between two consecutive trials (20–90 s, mean = 45 s).
  c. Run IDS protocol (Figure 2C).
    i. Fill the cylinder with water to a height of 21 cm from the base (Figure 2A).
      ***Note:*** This configuration ensures that the hind limbs of the mouse cannot touch the rescue plate (down position). Even if the tail makes contact, it cannot serve as a supporting point, avoiding interference with behavioral changes in mice. In addition, water temperature is measured manually before each session and maintained at 23°C.
    ii. Gently place a mouse on the rescue plate.
    iii. Initiate the IDS program and start video recording.
    iv. After each session, gently remove the mouse from the rescue plate. Gently dry it and place it in a warm cage for 1 h for recovery with free access to food and water. Mice are returned to their home-cages after recovery.
    v. Refill the cylinder with fresh water to maintain a clean and controlled experimental environment.
    vi. Repeat the same protocol on Days 2 and 3.

### Analyze the behavior

**Timing: variable**

**Figure 3:**
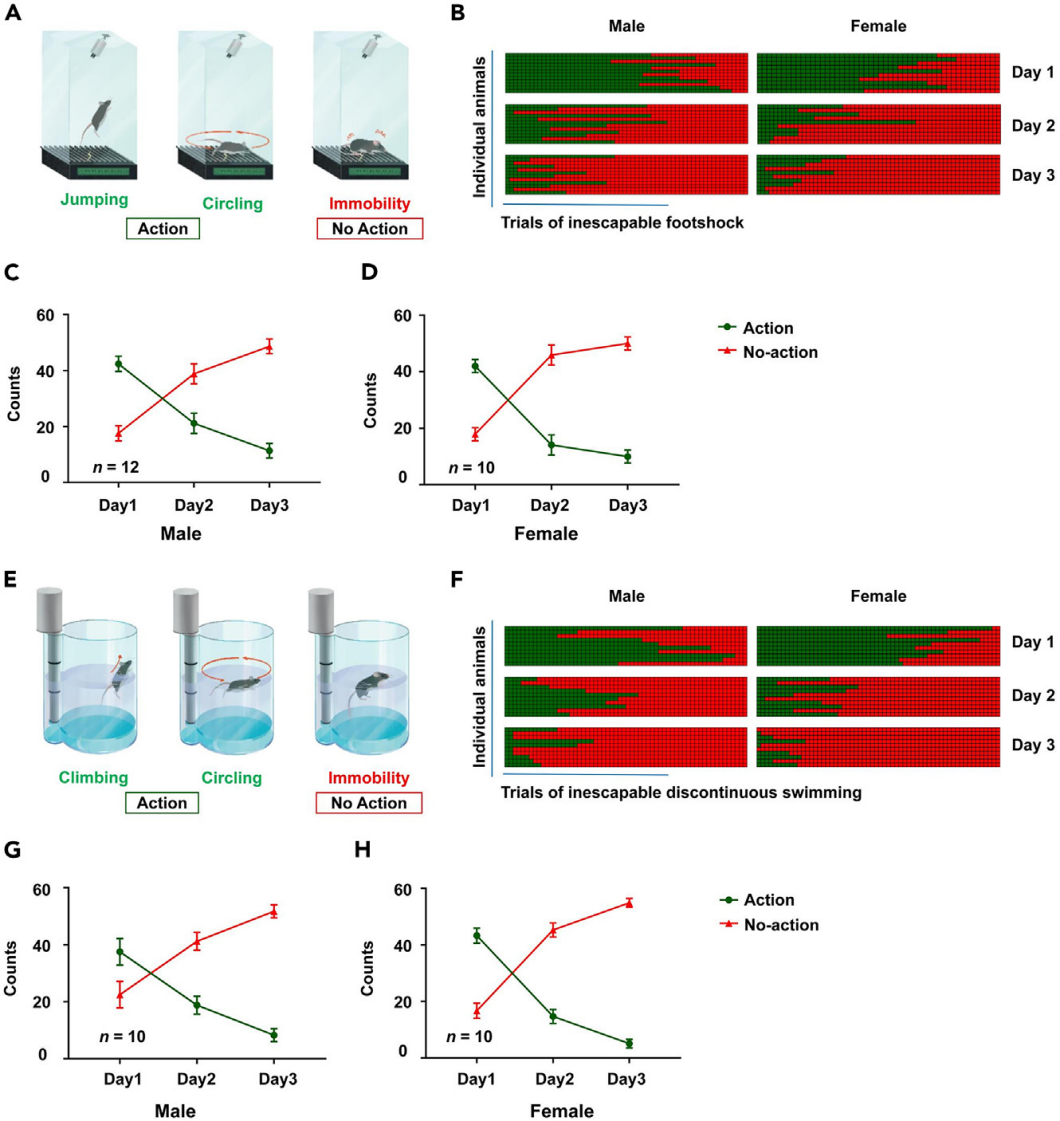
Analysis of the behavioral transition from action to no-action in IS and IDS models for male and female mice (A) Illustrative images of typical behaviors exhibited in the IS model. (B) Show the gradual transition from trials dominated by action behavior at the beginning to trials dominated by no-action in the IS model for males (n = 12) and females (n = 10). (C and D) Line charts depict the gradual transition from action to no-action over three-day sessions in the IS model in males (C) and females (D). (E) Illustrative images of typical behaviors exhibited in the IDS model. (F) The gradual transition from trials dominated by action behavior at the beginning to trials dominated by no-action in the IDS model for males and females (n = 10 per group). (G and H) Line charts depict the gradual transition from action to no-action over three-day sessions in the IDS model in males (G) and females (H). Data are presented as mean ± SEM. See also Methods video S2.

This section describes how to analyze the behavior of mice (male and female) in IS and IDS models (Figure 3 and Methods video S2).

3. Observe the videotaped sessions trial by trial.
4. Quantify the behavior of the animals in each trial during the 15 s of foot shock (in the IS model) or discontinuous swimming (in the IDS model) experiences.
5. Determine the dominant behavior for each trial (i.e., the behavior that takes up most of the time during the 15 s of each trial).
6. Define the behavior in the trial as action or no-action based on the dominant behavior. troubleshooting 1 and 2.
  a. Define any instance of jumping, running and circling behaviors as action in the IS model. Define immobility behavior or passive movement as no-action in the IS model (Figure 3A and Methods video S2).
  b. Define vertical (climbing), horizontal and circular swimming behaviors as action in the IDS model. Define immobility behavior or passive swimming as no-action in the IDS model (Figure 3E and Methods video S2).

**CRITICAL:** Passive swimming includes minimum movements necessary for maintaining nostrils above the water surface or spontaneous floating of the body on the water surface without active movement from two or more limbs.

7. Plot the number of action vs. no-action trials over the three days of prolonged uncontrollable experiences (Figure 3).

***Note:*** Our models demonstrate generalizability to both male and female mice as both exhibit similar patterns of behavioral transition over three days of uncontrollable experiences in both models (Figure 3).

Methods video S2. Different types of behavior exhibited during IS and IDS trials are related to step 6. In the IS model, jumping and circling/running are classified as action behavior, while immobility or passive movement is classified as no-action In the IDS model, climbing and circling are classified as action, and immobile or passive swimming is classified as no-action. The videos are adapted with permission from Li et al. (2023).^1^

### Make a mathematical analysis of the behavioral transition

**Timing: variable**

This section explains how to combine computational analysis with the obtained behavioral data to create a curve to assess behavioral transition mathematically. It helps determine the exact time at which behavioral transition occurs in individual mice. It also helps calculate the transition rate value “α,” which can be used for quantitative and statistical analyses.

***Note:*** We employ an exponential learning equation, a standard equation for tasks involving numerous repeated trials with binary outcomes (success or failure).^5^^,^^6^ Therefore, we utilize these equations to generate a mathematical model (Equation 4).

**Figure 4:**
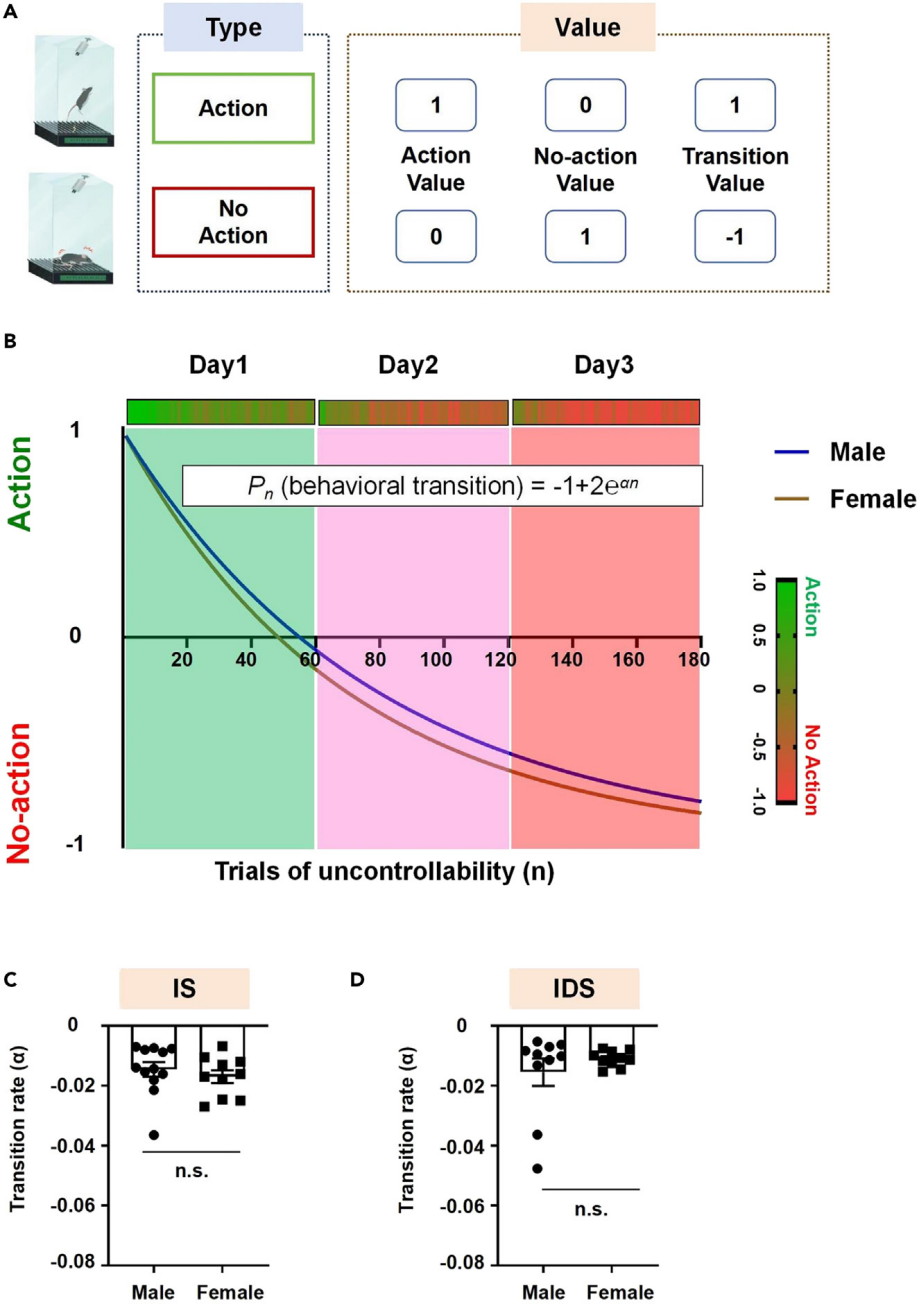
Mathematical model of behavioral transition (A) Illustrative example demonstrating the coding of mathematical values for different types of behavior. (B) The best fit line of IS and IDS datasets is after fitting the data from Figure 3 into Equation 4 for male and female mice. (C and D) Transition rate (α-value) derived from the transition curve of individual male and female mice in the IS (C) and IDS (D) models. α-value suggests that the models can be generalized to male and female mice. Data are presented as mean ± SEM. Unpaired *t*-test (n.s., not significant). See also Data S1.
9. Create two separate binary systems from the same data set for action and no-action. Start from the first trial:
  a. Generate “Action Value”.
  b. Generate “No-Action Value.”
  c. Generate the “Transition Value.”

***Note:*** Code the action binary representations as follows: give the trial a value of 1 if the mouse’s behavior is action on this trial. If the mouse’s behavior is not action, then give it a value of 0. Code the no-action binary representations as follows: give the trial a value of 1 if the mouse’s behavior is no-action on this trial. If the mouse’s behavior is not no-action, then give it a value of 0. Subtract every trial in the no-action code from the corresponding trial in the action code to generate the “Transition Value” according to the following equation: Transition Value = Action Value - No-Action Value. By now, the “Action Transition Value” is “1”; meanwhile, the “No-Action Transition Value” is “-1”.

8. Convert the action/no-action scores into binary representations (Figure 4A, Data S1).

10. Develop the final exponential learning equation.
  a. Give the original form of the exponential learning equation.
  b. Generate the “Action Value” equation.
  c. Generate the “No-Action Value” equation.
  d. Generate the “Transition Value” equation.

***Note:*** The original form of the exponential learning equation is given by Equation 1, where n is the number of trials, P0 is the initial behavioral performance, P∞ is the asymptotic behavioral performance, α is the transition rate coefficient, and Pn is the observed behavioral performance on trial n. For the “Action Value” derived above: P0 is 1, P∞ is 0, then the “Action Value” equation is given by Equation 2. For the “No-Action Value” derived above: P0 is 0, P∞ is 1, then the “No-Action Value” equation is given by Equation 3. The final behavioral transition equation can be similarly derived as we did for the “Transition Value;” by subtracting Equation 2 from Equation 3 to generate Equation 4.

(Equation 1) $${P_{n}=P_{\infty }-(P}_{\infty }-P_{0})e^{\alpha n}$$

(Equation 2) $$P_{n}=e^{\alpha n}$$

(Equation 3) $$P_{n}=1-e^{\alpha n}$$

(Equation 4) $$P_{n}=-1+{2e}^{\alpha n}$$

11. Fit the behavioral data (after converting them to “Action Transition Values” and “No-Action Transition Values” as described above) into Equation 4, generate the best fit line (by using GraphPad Prism or any other specialized software) and calculate the transition rate (α) for each mouse (Figures 4B–4D). troubleshooting 3.

***Note:*** A low α indicates a slow behavioral transition (favoring action), while a high α indicates a fast behavioral transition (favoring no-action). Employ this transition rate for statistical analyses related to the regulation of the behavioral transition. One can also statistically compare the action and no-action counts between the experimental groups on days 1, 2 and 3.

### Perform further validation experiments

**Timing: 13 days**

In our published study,^1^ we have presented robust evidence indicating that the behavioral transition from action to no action is not due to learned helplessness/anhedonia-like status, muscle fatigue, or pain desensitization. Furthermore, we demonstrate that antidepressant treatments cannot reverse the behavioral transition.

**Figure 5:**
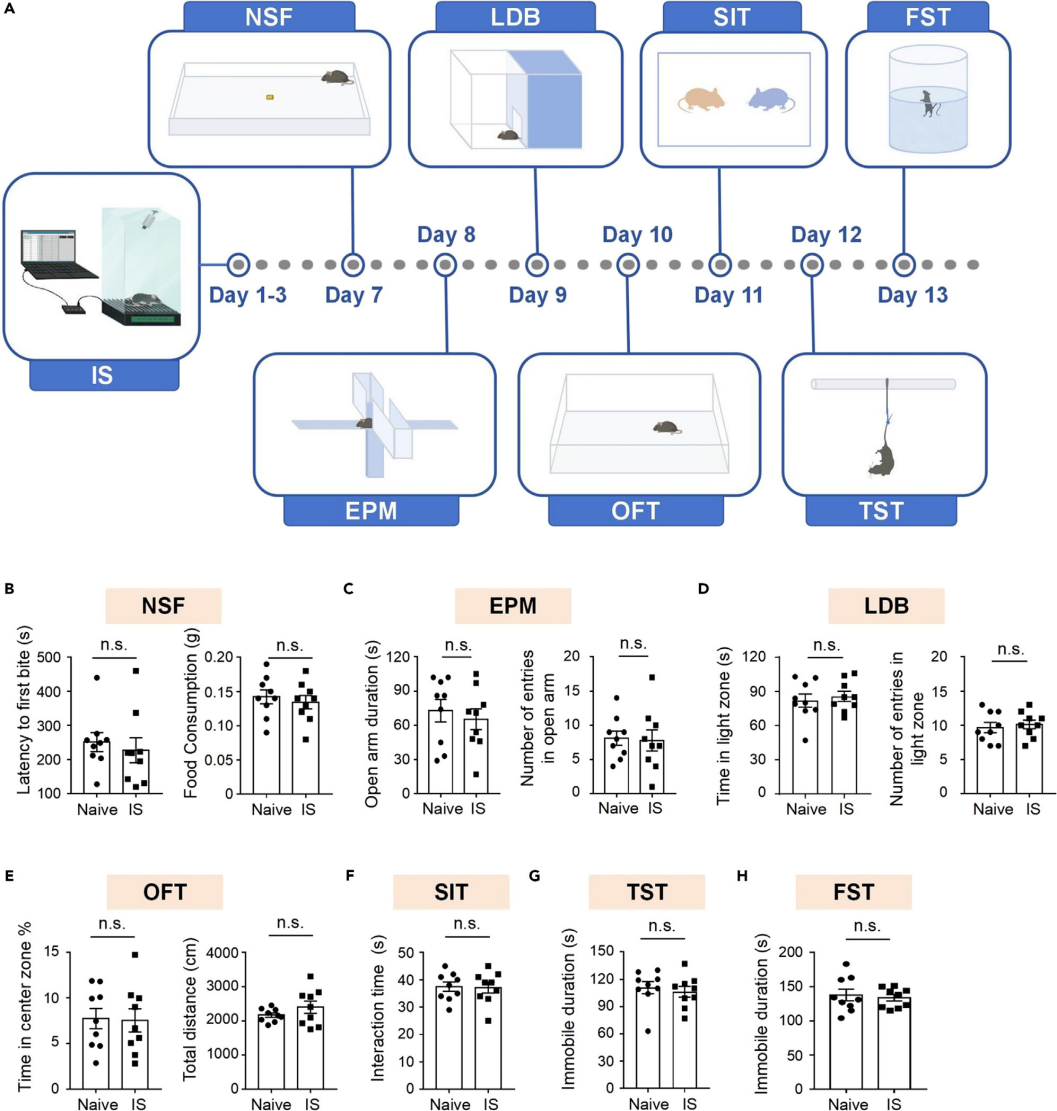
Further validation experiments for the IS model (A) Schematic Illustration depicting the experimental design of performing IS model followed by validation/control experiments that can be conducted using these models. (B–H) Behavioral results of naïve control mice and IS model exposed mice (n = 9 per group) in novelty suppressed feeding test (NSF, B), elevated plus maze test (EPM, C), light dark box test (LDB, D), open field test (OFT, E), social interaction test (SIT, F), tail suspension test (TST, G) and forced swimming test (FST, H). Data are presented as mean ± SEM. Unpaired *t*-test and unpaired Mann-Whitney test are used (n.s., not significant).

In this section, we further validate the IS model by demonstrating that the behavioral transition resulting from such traumatic, painful experiences is not associated with impairments in exploratory and social interaction functions, nor is it indicative of traumatic experiences related to depression- or anxiety-like behaviors. These experiments include an open field test (OFT, exploratory motor functions), social interaction test (SIT, social test), tail suspension test (TST) and forced swimming test (FST, two depression-like behavioral tests), novelty suppressed feeding (NSF) test, elevated plus maze (EPM) test, light-dark box (LDB) test (three anxiety-like behavioral tests) (Figure 5A). The results confirm that the behavioral transition is not linked to changes in locomotor activity, social behavior, or anxiety/depression-like behaviors (Figure 5).

It is unnecessary to conduct every experiment in the following section, but depending on your hypothesis, these may be helpful to control studies to help exclude alternative explanations and support your conclusions.

12. Expose the mouse into the IS session from day 1 to day 3 following the IS protocol (step 1).
13. Conduct the novelty suppressed feeding test (NSF) on day 7, using an open field box (50 × 50 × 50 cm) (Figures 5A and 5B).
  ***Note:*** Food deprivation is included in the 3-day interval between the IS experiment and the NSF test. If not starting with NSF, researchers can reduce the interval to a minimum of 1 day to ensure no acute effect after the three IS or IDS model sessions on subsequent validation and control behavioral tests. Finally, no changes in the animals' housing conditions are introduced during this period to avoid any potential interference with animal behavior. The NSF test has been used to assess anxiety-related behavior in animals and has become a practical paradigm for testing anxiolytic and antidepressant drugs.^7^
  a. Deprive the mouse of food for 24 h (on day 6) by removing the food from the feeding pellets area and ensuring no feeding pellets were hidden inside the cage.
  b. Place a small amount of highly palatable food in the center of the field.
  c. Place the mouse randomly in a corner facing the food and allow it to explore the field until the first feeding (or for a maximum test period of 10 min).
  d. Record the entire procedure using a video camera and the Limelight software.
  e. Analyze the videos to determine the latency to start feeding in a novel environment.
    ***Note:*** This behavioral readout is used as an indication of anxiety-like behavior. A high latency indicates high anxiety.
  f. Return the mouse to its home-cage.
  g. Provide highly palatable food for another 10 min immediately.
  h. Measure the amount of food consumed during this 10 min interval in the home-cage by weighing the food before and after this interval.
    ***Note:*** This is a control step to ensure that basic food consumption behavior in the home-cage remains normal.
  i. Return the food to the home-cage according to standard housing conditions.
14. Conduct the elevated plus maze test (EPM) on day 8 (Figures 5A and 5C).
  ***Note:*** The EPM device consists of two closed arms (30 × 6 × 20 cm, length × width × height), two open arms (30 × 6 cm, length × width), and a central platform (6 × 6 cm, length × width). The EPM is elevated at 50 cm above the ground. The EPM test is a widely adopted method for assessing anxiety-like behavior. It is based on the natural aversion of mice to the opened and elevated areas. It is an effective paradigm for testing anxiolytic and antidepressant drugs.^8^
  a. Place the mouse on the central platform gently, with its head facing the open arm direction, and leave it in the EPM for 5 min.
  b. Record the session using a camera positioned above the device, controlled by Limelight software.
  c. Analyze the videos to quantify the time spent exploring the open arms and the number of entries into the open arms.
    ***Note:*** These measures are used as indicators of anxiety-like behavior. Lower time and entries in open arms indicate higher anxiety.
15. Conduct the light dark box test (LDB) on day 9 (Figures 5A and 5D).
  ***Note:*** The LDB device consists of two parts: a bright chamber (15 × 20 × 25 cm, length × width × height) lit with solid light (150 lux) and a dark, smaller chamber (15 × 10 × 25 cm, length × width × height). The two chambers are connected through a small door (4 × 4 cm), allowing mice to shuttle between both chambers freely. The light/dark test is based on rodent’s innate fear of brightly illuminated areas. The test provides an effective method for detecting anxiolytic-like or anxiogenic-like status in mice.^9^
  a. Place the mouse in the light chamber gently.
  b. Record the total time spent in the light chamber and the number of entries during a 5 min test period.
    ***Note:*** Time/entries in the light chamber are used as indicators of anxiety-like behavior. Lower time/entries indicate higher anxiety.
16. Conduct the open field test (OFT) in a brightly lit polyvinyl chloride (PVC) box (50 × 50 × 50 cm) on day 10 (Figures 5A and 5E).
  ***Note:*** The OFT is a critical behavioral test for studying the neurobiological basis of motor exploratory activity, anxiety, and screening for novel anxiolytic drugs.^10^
  a. Place the mouse gently in the center of the open field and allow it to explore for 5 min freely.
  b. Record the session and analyze the videotapes for the total distance and time spent in the center using Limelight 4.0 software.
    ***Note:*** Total distance is a standard behavioral readout for locomotor and exploratory activity. Use the time in the center as an indicator of anxiety-like behavior. Lower time in the center indicates higher anxiety.
17. Conduct the social interaction test (SIT) in an open arena without bedding (25 × 25 × 30 cm, length × width × height) on day 11 (Figures 5A and 5F).
  ***Note:*** After the OFT session, we habituated the mice to the empty SIT arena for 10 min. On day 11, habituate the same mice again in the same empty arena for another 10 min. After these two habituation sessions, we expose the mice to the SIT session.
  a. Place the mouse gently into the same arena containing an unfamiliar mouse and let the mice interact freely.
  b. Videotape the session for 5 min test period.
  c. Analyze the videos and quantify the total social interaction time between the two mice initiated by the experimental mouse (not the unfamiliar mouse).
    ***Note:*** Social interaction behaviors include sniffing, grooming, chasing and climbing on each other.
18. Utilize a rod-equipped bracket for the tail suspension test (TST) on day 12 (Figures 5A and 5G).
  ***Note:*** This bracket features a fixed rod typically 55 cm above the table surface.
  a. Suspend the mouse from its tail by tapping the tail on the rod that is fixed on the bracket.
  b. Record the test session for 6 min.
  c. Quantify the immobility time of the mouse during the last 4 min.
    ***Note:*** Score immobility time when the mouse hangs passively without moving its paws.
19. Conduct the forced swimming test (FST) in a transparent cylinder (diameter: 14 cm, height: 30 cm) and maintain the temperature of water at 23°C on day 13 (Figures 5A and 5H).
  a. Fill the cylinder with water (the water level is 17.5 cm from the base).
    ***Note:*** The water depth should be sufficient to prevent the mouse from touching the bottom with its tail or hind limbs.
  b. Place the mouse gently into the cylinder.
  c. Record the test session for 6 min.
  d. Quantify the immobility time of the mouse during the last 4 min.
    ***Note:*** Score immobility time when the mouse is passively floating in the water without doing any active movements except that necessary to keep the nostrils above the water.
  e. Pick up the mouse, dry it gently and place it in a warm cage for 1 h to recover with food and water ad libitum after the test.
  f. Return the mouse to its home-cage.
    ***Note:*** The TST test and FST test are used to measure behavioral despair or “depression-like” behavior. These tests aid in screening potential antidepressant drugs and assessing other manipulations expected to affect depression-related behaviors.

## Expected outcomes

When uncontrollability is prolonged, human subjects gradually transition from an action-oriented cognitive state to no action-oriented cognitive state. Similarly, our models show that exposing mice to prolonged experiences with an uncontrollable outcome (repeated failure to escape) results in a gradual behavioral shift from action to no-action. We observed that on day 1, the mouse behavior was dominated by action (jumping and circling in the IS model, climbing and circling in the IDS model). Over time, the mouse exhibited a gradual behavioral transition to no-action (immobility in IS and IDS model, as shown in Figure 3 and Methods video S2). Therefore, we have developed two experimental mouse models representing behavioral transition during prolonged experiences with an uncontrollable outcome. These paradigms differ from endophenotypes associated with animal models of depression, anxiety, post-traumatic stress disorder (PTSD), anhedonia, and learned helplessness. These tractable, robust and reproducible models can be used to study neural mechanisms underlying give up-like/quitting-like behavior in the face of repeated failure.

It is worth noting that during any inescapable stressful experience, the key factors underlying the exhibition of depression-/learned helplessness-like behaviors are the intensity of the stressor and the time window during which the animal is exposed to the stressor. Standard learned helplessness protocols range from 180 trials of inescapable shocks in one day to 360 trials divided over 2 days.^11^^,^^12^ Our protocol comprises 60 trials of inescapable shocks per day. Therefore, after one and two days, we did not detect escape deficits,^1^ suggesting that behavioral transition occurs before any learned helplessness-like behavior is detected. Regarding the IDS model, our protocol is also different. In the standard FST protocol, the animal is left in water for 6 min (2 min adaptation, immobility time is measured during the last 4 min).^13^ Based on the first sign of robust immobility that is usually exhibited by control animals (Approximately 150–180 s), one can estimate that the mice are generally left in water for 270–300 s before they start exhibiting depression-like behavior (immobility). In our IDS mode, the animals are placed in water for 15 s only before they are left again in every trial. Thus, there is no time/chance/reason to develop behavioral despair. Therefore, in our models we have reduced the intensity of the stressors (amount vs. exposure time) such that it is not sufficient to induce learned helplessness-/depression-/anxiety-like behaviors, but adequate to induce behavioral transition from action to know action resembling give up-/quitting-like behavior.

## Quantification and statistical analysis

GraphPad Prism is used for data analysis. Shapiro-Wilk normality test is used. For statistical significance testing of normally distributed data, unpaired Student’s t-test is used. The nonparametric unpaired Mann-Whitney test is used if the data are not normally distributed. Statistical significance is defined as a p-value < 0.05.

## Limitations

Firstly, young and old subjects exhibit different responses during the same experience. The current protocols have not yet been tested in young and aging mice, potentially limiting our models' generalizability. However, here, we demonstrate the generalizability of our models to male and female mice (Figures 3 and 4).

Secondly, we aim to introduce novel behavioral models representing the transition from action to no-action when facing repeated failure. Our goal is to ensure that quantitative analysis of the behavior is feasible, reproducible, and easy to learn. Consequently, we employ a binary system to categorize the behavior as either action or no-action. On the other hand, it is crucial to identify a discrete time point at which the transition from action to on-action occurs. Therefore, a time-based quantitative description of behavior (e.g., percentage immobility over time) rather than the binary outcome would provide more detailed and precise descriptions. However, using the percentage of time (spent in action/no-action) is only practical if behavior monitoring can be automated. It is difficult for different experimenters to generate a consistent quantitative analysis of the same experiment by measuring the action/no-action time because it is hard (even impossible) for the experimenters to remain fully focused to watch the videos and precisely quantify the time of each type of behavior for 1 h per mouse, up to 10 mice per group, up to 4 groups in specific experiments (i.e., up to 40 h for one day, each model lasts for 3 days). Hence, we strongly recommend using our binary action/no-action system to maintain the feasibility, robustness, reproducibility, and ease of learning in quantitative behavior analysis.

Thirdly, in our mathematical model (Equation 4), we considered -1 as a representative value of no-action in the binary system we used. That means we presumed that the mice reached the asymptotic level of learning according to the exponential learning equation we adopted. However, actual behavioral data indicate that the mice did not reach that asymptotic level. All male and female mice from both models reached an average of -0.815, not -1. Further exposure of the animals to IS or IDS experiences (e.g., day 4, 5…) will result in reaching the asymptotic level of no-action. However, the animals are more likely to exhibit learned helplessness-/depression-like behavior after such very prolonged exposure to uncontrollable experiences. This will change the model’s nature as animal models to study brain mechanisms of give up/quitting/failure-related behavior. Future studies are warranted to optimize a mathematical model for the behavioral transition.

## Troubleshooting

### Problem 1

In the IS model, a very small number of mice (chance is approximately 1 in 15) might learn to escape the electrical foot shock by standing and holding onto one bar of the metal grid. This could influence the behavioral readout by delaying the behavioral transition (related to Step 6a).

### Potential solution

• If the mouse stands or holds onto only one metal grid to avoid the foot shock in the IS model, the experimenter should use a long pole to give it a gentle push to move. This will help ensure that the mouse receives the foot shock.
• If the mouse continues to exhibit this behavior more than 5 times, it should be removed and excluded.

### Problem 2

In the IDS model, repeated up/down movements from the mouse and the rescue plate will cause strong turbulent flow and significant irregular fluctuations in the water surface. This turbulence will force the animal to struggle to keep its nostrils above the water, thus influencing the behavioral readout by delaying the behavioral transition (related to Step 6b).

### Potential solution

• The experimenter should adjust the descending speed of the robotic arm (i.e., slow it down to minimize irregular fluctuations in the water surface).
• The experimenter can slightly loosen the connection between the robotic arm and the rescue plate. This will increase the rescue plate’s vibration during the robotic arm’s motion, calming down the water and reducing the surface turbulence.

### Problem 3

In our mathematical model, we presumed that the mice reached the asymptotic level of -1 according to the exponential learning equation, although the mice did not. Therefore, the mathematical model still requires further optimization in the future (related to Step 11).

### Potential solution

• Until further optimization of the mathematical model is achieved, one can use the current model to derive the α-value and utilize it for statistical analysis, given that necessary normality and distribution tests are conducted.
• One can use the original scores of action/no-action (on days 1, 2, and 3) and compare them directly between different experimental groups.

## Resource availability

### Lead contact

Further information and requests for resources and reagents should be directed to and will be fulfilled by the lead contact, Nashat Abumaria (Abumaria@fudan.edu.cn).

### Technical contact

Questions about the technical specifics of performing the protocol should be directed to and will be answered by the technical contact, Nashat Abumaria (Abumaria@fudan.edu.cn).

### Materials availability

This study did not generate new unique reagents. However, one new behavioral device (IDS, China patent, 2023) was generated. All necessary IDS device support will be provided upon reasonable request and after signing proper agreements with the lead contact.

### Data and code availability

This study did not analyze/generate any new unique data and codes.

## Supplemental information

Data S1. Action value from all male and female mice from trials from both models, related to step 8 and Figure 4
